# Application of spectrophores™ to map vendor chemical space using self-organising maps

**DOI:** 10.1186/1758-2946-3-S1-P7

**Published:** 2011-04-19

**Authors:** G Thijs, W Langenaeker, H De Winter

**Affiliations:** 1 Silicos NV, Diepenbeek, 3590, Belgium

## 

A Spectrophore™ is a one-dimensional descriptor that describes the three-dimensional molecular field surrounding a molecule generated by a given set of atomic properties. In a typical application, Spectrophores™ are calculated from the molecular shape in combination with the electrostatic, lipophilic, softness, and hardness potential surrounding the molecules. Given that molecules with similar three-dimensional properties have similar Spectrophores™ and the real-valued nature of Spectrophores™, they are very well suited to deploy in various virtual screening models. Spectrophores™ have been recently released in the open source domain and have been added as a descriptor in the Open Babel C++ API.

To illustrate the capabilities of Spectrophores™ for virtual screening and clustering, a large self-organising map (SOM) was trained from 8.6 million commercially available compounds as a representation of the vendor chemical space. To train a SOM from such a large collection of compounds, a specially tailored algorithm was developed. The resulting SOM clearly shows that the vendor chemical space can be mapped in a meaningful organization based on its Spectrophores™ representation and that it handles compounds beyond their topological similarity. The SOM has been applied in three different problems, including the overlay of vendor databases, the comparison of compounds with different pharmacological profiles, and to sample drug-like screening libraries.

